# Experimental colonization with *H. hepaticus*, *S. aureus* and *R. pneumotropicus* does not influence the metabolic response to high-fat diet or incretin-analogues in wildtype SOPF mice

**DOI:** 10.1016/j.molmet.2024.101992

**Published:** 2024-07-15

**Authors:** Margit Wunderlich, Manuel Miller, Bärbel Ritter, Ronan Le Gleut, Hannah Marchi, Monir Majzoub-Altweck, Patrick J. Knerr, Jonathan D. Douros, Timo D. Müller, Markus Brielmeier

**Affiliations:** 1 Core Facility Laboratory Animal Services, Helmholtz Munich, Germany; 2 Core Facility Statistical Consulting, Helmholtz Munich, Germany; 3 Institute of Veterinary Pathology, Ludwig-Maximilians-University Munich (LMU), Germany; 4 Indiana Biosciences Research Institute, Indianapolis, IN, USA; 5 Institute for Diabetes and Obesity, Helmholtz Munich, Germany, and German Center for Diabetes Research, DZD, and Walther-Straub Institute for Pharmacology and Toxicology, Ludwig-Maximilians-University Munich (LMU), Germany; 6 Faculty of Business Administration and Economics, Bielefeld University, Germany

**Keywords:** Diet-induced obesity model, Type 2 diabetes, C57BL/6J, *Helicobacter hepaticus*, *Rodentibacter pneumotropicus*, *Staphylococcus aureus*

## Abstract

**Objectives:**

We here assessed whether typical pathogens of laboratory mice affect the development of diet-induced obesity and glucose intolerance, and whether colonization affects the efficacy of the GLP-1R agonist liraglutide and of the GLP-1/GIP co-agonist MAR709 to treat obesity and diabetes.

**Methods:**

Male C57BL/6J mice were experimentally infected with *Helicobacter hepaticus, Rodentibacter pneumotropicus* and *Staphylococcus aureus* and compared to a group of uninfected specific and opportunistic pathogen free (SOPF) mice. The development of diet-induced obesity and glucose intolerance was monitored over a period of 26 weeks. To study the influence of pathogens on drug treatment, mice were then subjected for 6 days daily treatment with either the GLP-1 receptor agonist liraglutide or the GLP-1/GIP co-agonist MAR709.

**Results:**

Colonized mice did not differ from SOPF controls regarding HFD-induced body weight gain, food intake, body composition, glycemic control, or responsiveness to treatment with liraglutide or the GLP-1/GIP co-agonist MAR709.

**Conclusions:**

We conclude that the occurrence of *H. hepaticus*, *R. pneumotropicus* and *S. aureus* does neither affect the development of diet-induced obesity or type 2 diabetes, nor the efficacy of GLP-1-based drugs to decrease body weight and to improve glucose control in mice.

## Highlights

• Bacteria do not affect development of diet-induced obesity or glucose intolerance in mice.
• Bacteria do not compromise the beneficial effect of incretin-analogues on body weight or glucose control.
• Macro- and microscopic changes do not differ between uninfected and infected groups.

## Abbreviations

AUC: area under the curve
DIO: diet-induced obesity
ELISA: enzyme-linked immunosorbent assay
EDTA: ethylenediaminetetraacetic acid
GLP-1: glucagon-like peptide-1
GIP: glucose-dependent insulinotropic polypeptide
HE: hematoxylin-eosin
HFD: high-fat diet
*H. hepaticus*: *Helicobacter hepaticus*
IL-10: interleukin 10
ipGTT: intraperitoneal glucose tolerance test
IVC: individually ventilated cage
MALDI-TOF: matrix assisted laser desorption ionization – time of flight
PBS: phosphate-buffered saline
*R. pneumotropicus*: *Rodentibacter pneumotropicus*
*S. aureus*: *Staphylococcus aureus*
SEM: standard error of the mean
SOPF: specific and opportunistic pathogen free
TAG: triacylglyceride
T2DM: type 2 diabetes mellitus

## 1 Introduction

Type 2 diabetes mellitus (T2DM) is a complex metabolic disease that affects 537 million adults worldwide in 2021 [1]. By 2045, the number of patients is estimated to increase to more than 700 million people [2,3]. Therefore, it is important to develop new drugs and compounds with higher efficacy for better treatment options. Murine models are important tools to study and understand obesity, T2DM and the mechanisms by which drugs affect energy and glucose metabolism. The most widely used mouse strain to study lifestyle-induced obesity is the C57BL/6J mouse, which upon feeding a high-fat diet (HFD), develops diet-induced obesity (DIO) and many associated comorbidities, including glucose intolerance, insulin resistance and hepatosteatosis [[4], [5], [6], [7], [8]]. The development of glucose intolerance in DIO mice is largely comparable to the pathogenesis in humans [9]. Factors implicated in the development of human obesity include genetic predisposition, unhealthy diet, and physical inactivity. These factors lead to obesity, high blood glucose levels and associated diseases, which are summarized as the metabolic syndrome. Diabetic patients also show an increased susceptibility to infection and are more frequently colonized and infected with pathogenic bacteria [10,11]. In the animal facility, standardization and verification of hygiene is of paramount importance for data comparability and interpretation. Although certain pathogens are excluded from animal husbandry, mice can be colonized with opportunistic or pathogenic bacteria. These bacteria usually do not cause clinical symptoms in immunocompetent animals and are therefore often accepted. However, they can still influence experimental results [[12], [13], [14], [15]]. Contrary to standardization, greater bacterial variability may increase the transferability of test results. For the study of the immune system, it has been described that experiments are more meaningful when laboratory mice are colonized with microorganisms from wild and pet mice. They received a better immune system more similar to that of adult humans [16,17]. Taken together, this raises the question of whether multi-colonized mouse models for diabetes research alter and might even improve the translation of results to humans compared to pathogen-free mice. In this study, we aimed to assess whether typical bacteria found in laboratory animal facilities influence the development of diet-induced obesity, glucose intolerance and the efficacy of best-in-class drugs to treat obesity and diabetes. Bacteria with high prevalence in laboratory mice are *Helicobacter hepaticus*, *Rodentibacter pneumotropicus* and *Staphylococcus aureus* [15,18,19]. These bacteria colonize the skin, mucosa or gut but rarely cause clinical signs in immunocompetent healthy mice [14,[20], [21], [22], [23], [24], [25], [26]]. *S. aureus* is a gram-positive bacterium that colonizes the skin and mucosa of the respiratory and gastrointestinal tracts. Clinically, it can present as dermatitis, wound infection, or abscess [12,14,23,27]. Another bacterium that inhabits the mucosa of the respiratory tract is *R. pneumotropicus* (*Pasteurellacea* family). It can also be isolated from the urogenital tract and from the intestine and is associated with pneumonia, inflammation of the eye or urogenital tract. *Rodentibacter* is gram-negative and host-specific to rodents [12,14,28,29]. The typical *Helicobacter* species for mice is *H. hepaticus.* This gram-negative, microaerophilic bacterium colonizes the liver and intestine of mice, especially the cecum. It can cause inflammation, necrosis, and carcinoma [14,24,30]. *H. hepaticus* colonization also alters the gut microbiome [31]. Increased rates of infection with *S. aureus* and *Helicobacter* species have been reported in obese and diabetic patients [11,[32], [33], [34], [35]]. In overweight children, the prevalence of *S. aureus* in the gut was increased [36]. *Pasteurellacea* species are zoonotic agents that can cause clinical signs in immunocompromised individuals. Diabetes mellitus is considered as a risk factor for the development of pasteurellosis [37]. Therefore, these bacteria are not only relevant in laboratory animal husbandry but are also common comorbidities in diabetic patients. An impact of the intestinal flora on obesity and type 2 diabetes development is known [[38], [39], [40], [41]]. The transplantation of microbiota from lean to obese mice or the immunization with ileum microbiota can increase insulin sensitivity [42,43]. The intestinal flora can also change drug efficacy [44]. Diabetes medication and gut microbiome influence each other, e.g. metformin or liraglutide [[45], [46], [47], [48], [49], [50], [51]]. To evaluate the influence of infectious agents in the predisposition to develop obesity and glucose intolerance, we monitored body weight development and glucose levels of mice colonized with *H. hepaticus*, *R. pneumotropicus* and *S. aureus* vs. non-colonized mice over 26 weeks of HFD feeding. Subsequently, we assessed whether the infection status had an impact on the metabolic efficacy of best-in-class drugs to treat obesity and diabetes, namely the GLP-1 receptor agonist liraglutide and the GLP-1/GIP co-agonist MAR709. Both of these drugs have been shown in previous studies to profoundly improve body weight and glucose control in C57BL/6J DIO mice [[52], [53], [54]].

## 2 Material and methods

### 2.1 Animals and model

For this project, the inbred mouse strain C57BL/6J was used as a DIO model. C57BL/6J is genetically well characterized and widely used for type 2 diabetes research due to its predisposition to diet-induced obesity and glucose intolerance [4,[55], [56], [57]]. Female mice are not suitable because they do not develop glucose intolerance, insulin resistance or hyperinsulinemia [58].

Male C57BL/6J mice were purchased from Charles River Laboratories (Charles River Germany GmbH & Co KG, Sulzfeld, Germany) at 3–4 weeks of age with health status specific and opportunistic pathogen free (SOPF) and proven to be free from the bacteria of interest. Mice were housed in individually ventilated cages (IVC; GM 500, Tecniplast, Buggugiate, Italy) with a maximum cage density of four mice per cage. The light/dark cycle was 12h/12h, the temperature was 22 ± 2 °C and the air humidity was 55 ± 10%, according to Directive 2010/63/EU. Cages were enriched with autoclaved nesting material (crinklets and paper towels), mouse house or tunnel and chewing wood. All mice were fed ad libitum throughout the experiment with a high-fat diet (58% kJ energy from fat and sucrose; D12331; Research Diets, New Brunswick, USA) and had free access to sterile filtered tab water.

The experiment was conducted in strict accordance with the EU Directive 2010/63/EU on animal experimentation and national law and was approved by the local government under reference number ROB-55.2-2532.Vet_02-20-150.

### 2.2 Bacteria and infection

Infection and subsequent colonization were successfully tested in a preliminary experiment with a small number of animals. The bacteria were selected based on their prevalence in animal husbandry and their relevance to diabetic patients [11,13].

At the beginning of the experiment, half of the animals were orally infected with a bacterial solution containing three different species. The group constellation of colonized mice was changed, as all mice within three cages remained negative for *H. hepaticus* after experimental infection. In addition, four animals were orally re-infected with *S. aureus* as they were negative in the PCR. Afterwards, a stable colonization was achieved in all infected animals for all three bacteria until the end of the experiment.

*H. hepaticus* was obtained from the Leibniz Institute DSMZ - German Collection of Microorganisms and Cell Cultures GmbH; DSM No.: 22909. *R. pneumotropicus* was obtained from the Leibniz Institute DSMZ - German Collection of Microorganisms and Cell Cultures GmbH; DSM No.: 21403. *S. aureus* was isolated from the animal husbandry, the strain was confirmed by MALDI-TOF. Bacteria were cultured on Columbia blood agar base with 5% sheep blood (VWR, Leuven, Belgium) as overnight cultures or obtained as an active culture directly from the DSMZ. For the infection solution, each bacterial culture on blood agar was dissolved in 3 mL of phosphate-buffered saline (PBS) and the cell number was counted under a microscope (Axioplan 2 imaging, Jena, Zeiss; phase contrast; objective 40:1) using a counting chamber (Neubauer chamber improved, chamber depth 0.02 mm). *H. hepaticus* was obtained as a live culture and used directly. The bacterial solution for each animal contained an infectious dose of 1 × 10^8^ organisms for each bacterium or 1 × 10^7^ organisms for *H. hepaticus* in a total volume of 75 μL and was administered by oral gavage. Half of the mice remained clean with SOPF status and received PBS in a volume of 75 μL to obtain the same procedures for all animals. To ensure the infection status, animals were housed in IVC cages and handled in a strict order, first clean SOPF mice and then infected mice. Infection status was assessed every two weeks in stool samples by real-time PCR for each mouse individually. Low copy numbers for *R. pneumotropicus* and *S. aureus* suggest that the main colonization site is not the gastrointestinal tract. This was confirmed by culturing throat and preputial swabs with high bacterial growth in a preliminary colonization test. For the purposes of this study, a positive PCR result in stool samples was deemed sufficient evidence of colonization. Analyses were performed using Rotor Gene Q with software 2.1 (Qiagen, Hilden, Germany). Real-time PCR sequences and protocols were adapted from literature. For *H. hepaticus*, primers and probes were adopted from Fischer [59]. The thermal cycling conditions were adapted: Initial denaturation at 95 °C for 15 min, 45 cycles of denaturation at 95 °C for 15 s, and each cycle followed by primer annealing and extension at 60 °C for 60 s. For *S. aureus*, PCR assay 1, SA442-probe 1 and protocol were used from Nijhuis et al. [60]. For *R. pneumotropicus,* primers and probes from Dole et al. were adopted [61]. The thermal cycling conditions were adapted similarly to *H. hepaticus*. Primers and probes were purchased from metabion international AG, Planegg, Germany. Reporter dyes 6-Fam and BHQ-1 were used for *S. aureus* and *R. pneumotropicus* respectively, and TAMRA was used as a quencher for *H. hepaticus*.

### 2.3 Drugs

After 26 weeks of feeding with a HFD, mice were divided into 6 groups (2 infection statuses and 3 treatments) with similar body weight, fat, and lean mass (n = 8–9). All mice were treated with either liraglutide (50 nmol/kg, GLP-1-agonist, [62]), MAR709 (10 nmol/kg, GLP-1/GIP-agonist, [53]) or phosphate-buffered saline (PBS) as a negative control (vehicle). Peptide were prepared according to previously reported methods [63]. Treatments were administered by daily subcutaneous injection at a volume of 5 μL per gram of body weight for 6 days, in the afternoon.

### 2.4 Metabolic parameters

Body weight of each mouse was measured weekly during the feeding stage or daily during the treatment week to demonstrate body weight development. Food intake of each cage was measured on a weekly basis during the feeding stage and daily during the treatment week. It was calculated by weighing the food rack before and after feeding, divided by the number of animals per cage and by the number of days. For statistical analysis, cages with food shredding mice were excluded. Blood samples were collected from each mouse every four weeks during the feeding stage. For blood sampling, the lateral tail vein was punctured, and glucose was measured directly using a handheld glucometer (FreeStyle Libre, Abbott GmbH, Wiesbaden, Germany). Whole blood samples were collected in EDTA-coated tubes and immediately placed on ice. To obtain plasma, blood samples were centrifuged at 5000 rpm for 10 min at 4 °C and stored at −20 °C. ELISA and enzymatic assays were performed in combination with a photometer (Varioskan LUX plate reader, Fisher Scientific GmbH, Schwerte, Germany and SkanIt software 5.0 for microplate readers). Insulin levels were determined using ALPCO, Mouse Ultrasensitive Insulin ELISA, New Hampshire, USA. Total cholesterol levels were measured using Fisher Diagnostics, Infinity Cholesterol Liquid Stable Reagent Kit, Horn, Netherlands. However, the last measurement could not be analyzed because the enzyme kit was no longer available from the manufacturer. Triglyceride levels were determined using FUJIFILM Wako Shibayagi Corporation, LabAssay Triglyceride, Gunma, Japan. In some blood samples, there was not enough blood to evaluate all parameters. Outliers and values outside the sensitivity range for each kit were excluded. The last blood sample at the end of the experiment could not be evaluated due to partial clotting of the samples.

### 2.5 Body composition (EchoMRI)

Body composition was analyzed before and after the treatment using a magnetic resonance whole body composition analyzer (EchoMRI-100H, Echo Medical Systems, Houston, Texas, USA) to determine changes in fat and lean mass.

### 2.6 Intraperitoneal glucose tolerance test (ipGTT)

IpGTT was performed after six days of treatment. Glucose was measured directly with a handheld glucometer (FreeStyle Libre, Abbott GmbH, Wiesbaden, Germany) from 6 h fasted mice as a baseline. Subsequently, 1.75 g glucose per kg body weight was injected intraperitoneally and glucose levels were measured 15 min, 30 min, 60 min and 120 min after glucose administration.

### 2.7 Histology

Mice were sacrificed by anesthetic overdose (250 mg/kg ketamine + 10 mg/kg xylazine) and cervical dislocation. Lung, kidney, liver, pancreas, spleen, stomach, duodenum, cecum, and colon were examined blinded by a pathologist for inflammation, bacterial presence, or other abnormalities. For this purpose, organs were preserved in formalin solution (neutral buffered 10%), dehydrated, and embedded in paraffin. Histological sections of 2–3 μm thickness were prepared and stained with hematoxylin and eosin (HE) or Giemsa. Slides were evaluated using a bright field microscope. Macrovesicular steatosis of the liver was evaluated according to Brunt et al. and Kleiner et al. [64,65]. Some intestinal samples could not be processed for histology because they were too small to fit into the embedding cassette.

### 2.8 Statistical analysis

Data are presented as ± standard error of the mean (SEM). Statistical analyses and graphs were performed using GraphPad Prism 9.5.0. Data were tested for normal distribution using the Shapiro–Wilk test. For normally distributed data, Welch-tests and post-hoc Bonferroni correction for multiple testing were used. Non-normally distributed data were analyzed using Mann-Whitney-U tests and Bonferroni-Dunn post hoc analyses. For small sample sizes, where normality cannot be checked, multiple unpaired t-tests and Bonferroni-Dunn post hoc analyses were performed. For fat and lean mass changes as well as ipGTT area under the curve (AUC) assessment, one-way ANOVA followed by Bonferroni post hoc test were performed, and for non-normally distributed data Kruskal–Wallis test followed by Dunn post hoc test were performed. Chi-square test was performed for the assessment of liver steatosis. Differences were considered significant when adjusted p < 0.05. P-values from post hoc tests are presented and rounded to three decimal places. The non-rejection of the null hypothesis of a statistical test (p ≥ 0.05) is not a proof of the absence of difference between the groups under study. Therefore, we increased the number of animals to be able to detect existing differences in pre-diabetes or drug efficacy caused by bacteria with a probability of 90% (power of 0.9).

## 3 Results

### 3.1 DIO mice colonized with highly prevalent opportunistic bacteria are suitable as a model for pre-diabetes

**Figure 1:**
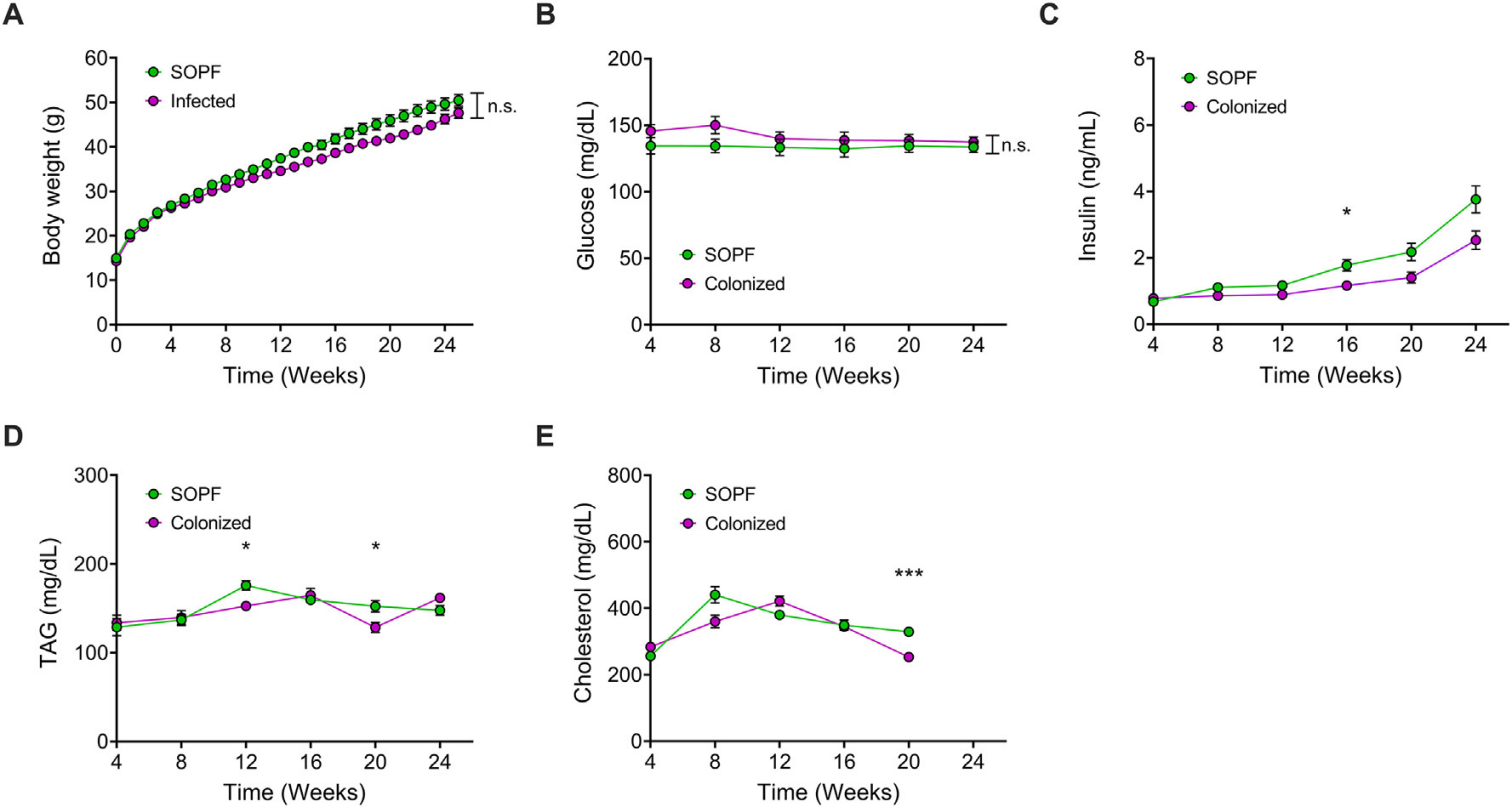
**Pre-diabetes development under HFD feeding.** A Body weight development, n = 26–27; B Blood glucose, n = 26–27; C Insulin, n = 17–26; D TAG, n = 18–26; E Cholesterol; n = 18–26, p < 0.05∗, p < 0.01∗∗, and p < 0.001∗∗∗, n.s. = not significant.

**Figure 4:**
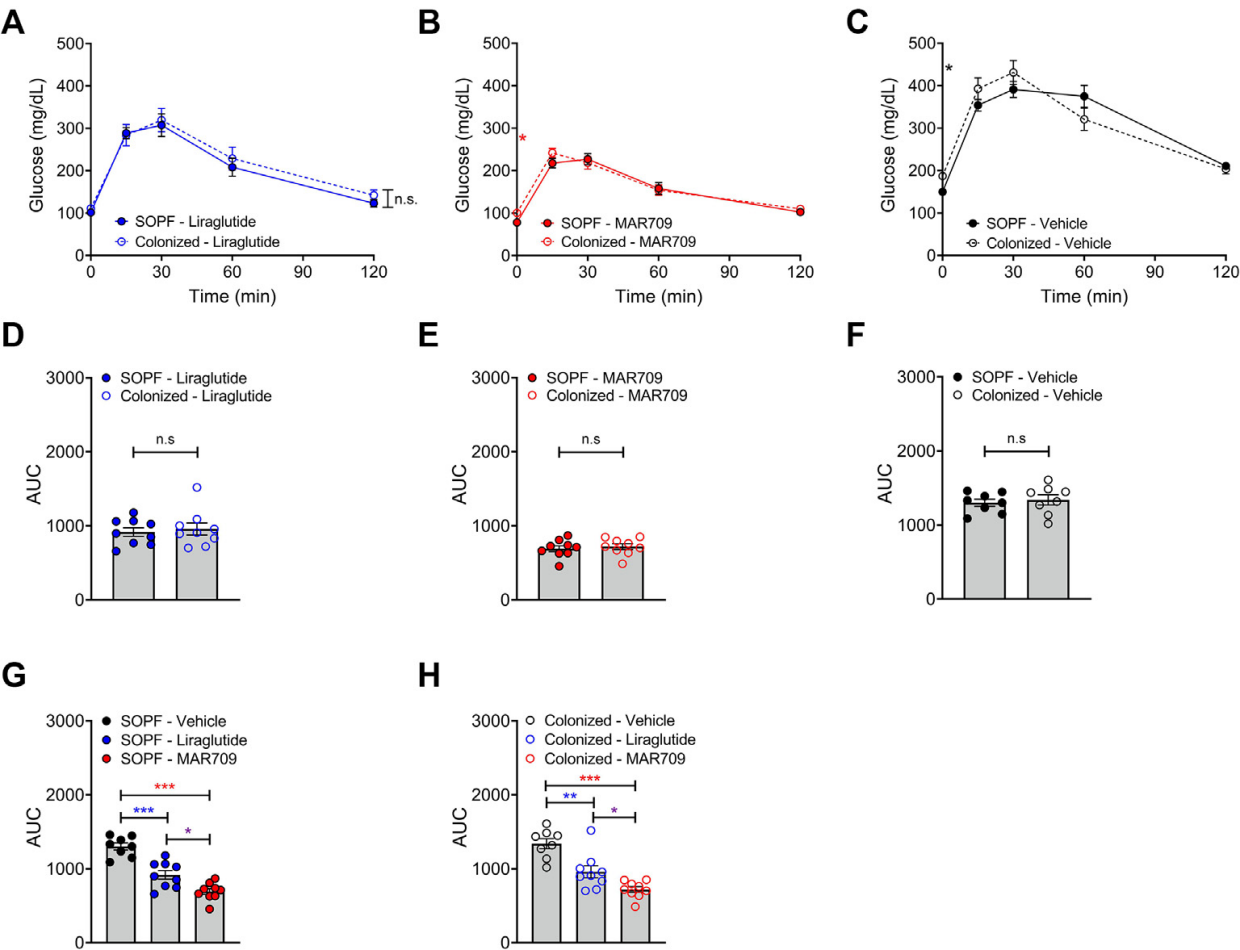
**Glucose tolerance after 6 h fasting.** A ipGTT liraglutide; B ipGTT MAR709; C ipGTT vehicle; D AUC liraglutide; E AUC MAR709; F AUC vehicle; G AUC SOPF mice; H AUC colonized mice; n = 8–9, p < 0.05∗, p < 0.01∗∗, and p < 0.001∗∗∗, n.s. = not significant.

Experimentally infected mice developed a stable colonization with all three bacteria (Table 1), non-infected SOPF mice showed negative results in the real-time PCR until the end of experiment. Colonized mice did not differ from non-colonized (SOPF) mice in terms of weight gain on HFD (Figure 1A). There were also no differences in food intake (Table 2). Blood glucose remained at the same level throughout the experiment, no hyperglycemia was observed (Figure 1B). Insulin levels increased in both groups, consistent with the development of insulin resistance during the progression of obesity. Nonetheless, insulin levels were slightly lower in colonized mice compared to non-colonized controls. Although insulin levels were slightly lower in colonized animals relative to the non-colonized controls (Figure 1C), no differences were observed in glucose tolerance (Figure 4A–C), hence indicating that the slight changes in insulin did not translate into changes in glucose control. There was a slight increase in triglycerides (TAG). Statistically significant differences were found at week 12 and week 20 with p = 0.011 and p = 0.042, respectively (Figure 1D). Nevertheless, the two curves are oscillating close to each other, showing that no biologically meaningful differences are observed between the two hygiene groups. Total cholesterol levels are similar in both groups, fluctuating upwards and downwards. There was a statistically significant difference between the two groups at week 20 with p < 0.001 (Figure 1E).

**Table 1:** Average copies per μl by real-time PCR of the colonized group.

	*H. hepaticus*	*R. pneumotropicus*	*S. aureus*
Before infection	0	0	0
After infection^a^	1.16 × 10^6^	126	10
Before treatment	8.76 × 10^5^	85	5
After treatment	2.60 × 10^5^	188	8

a Stable colonization after experimental infection as well as group re-constellation and oral re-infection.

**Table 2:** Food intake pre-diabetes development.

	SOPF	Colonized
Week 24	2.7 g ± 0.05	2.7 g ± 0.07
Week 25	2.6 g ± 0.07	2.7 g ± 0.06
Week 26	2.7 g ± 0.14	2.8 g ± 0.07

### 3.2 Treatment effects of incretin-analogues were not altered by bacterial colonization, MAR709 shows greater effects on glucose tolerance

**Figure 2:**
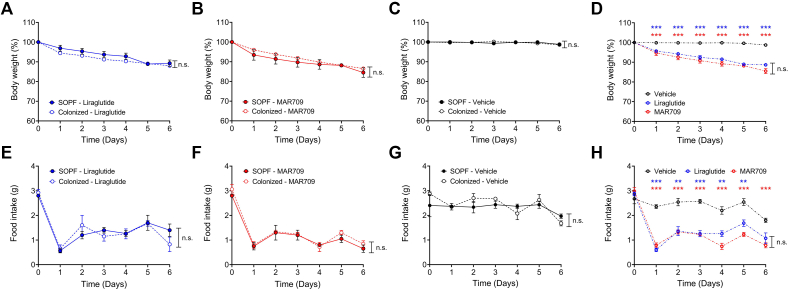
**Body weight and food intake under treatment with incretin-analogues.** A Body weight (%) liraglutide; B Body weight (%) MAR709; C Body weight (%) vehicle; D Body weight (%) grouped; E Food intake (g) liraglutide; F Food intake (g) MAR709; G Food intake (g) vehicle; H Food intake (g) grouped; n = 8–9, p < 0.05∗, p < 0.01∗∗, and p < 0.001∗∗∗, n.s. = not significant.

**Figure 3:**
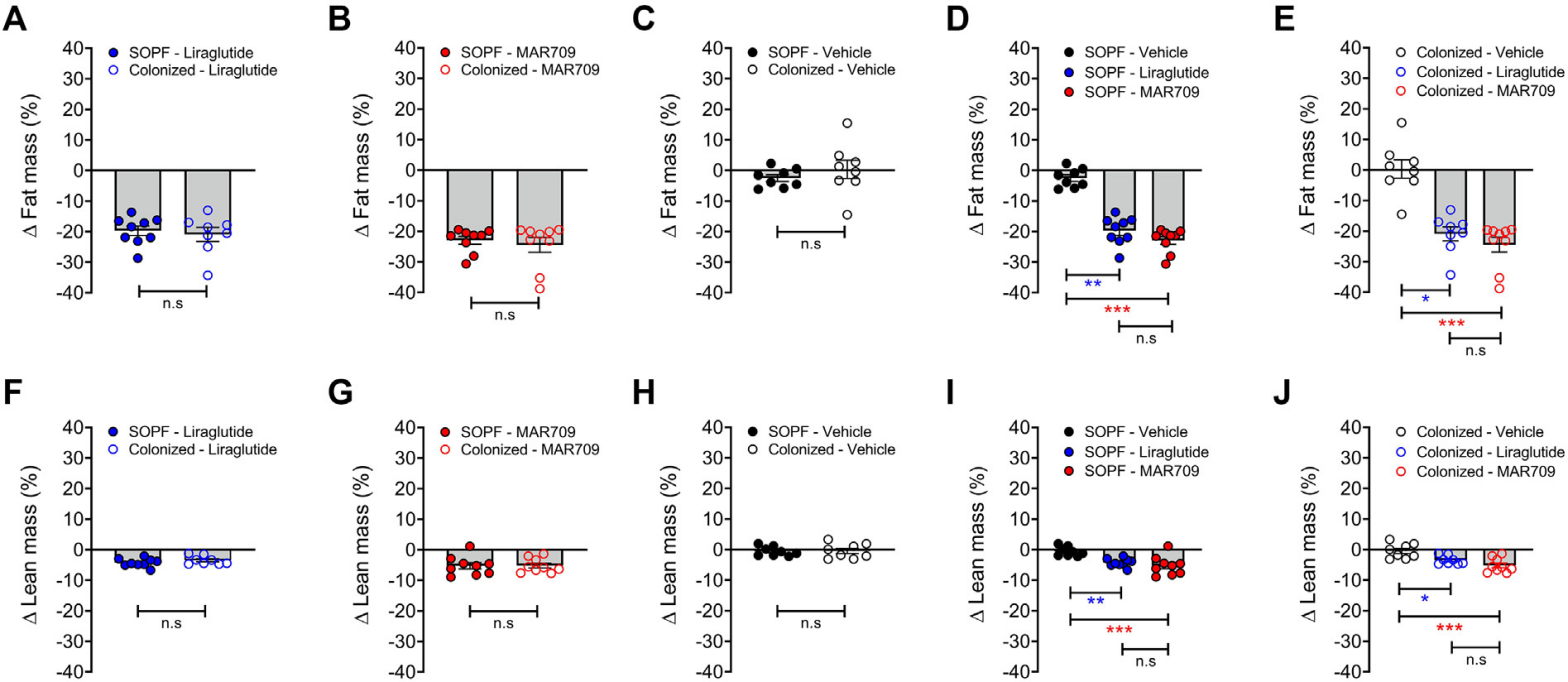
**Fat mass and lean mass under treatment with incretin-analogues.** A Fat mass (%) liraglutide; B Fat mass (%) MAR709; C Fat mass (%) vehicle; D Fat mass (%) SOPF mice; E Fat mass (%) colonized mice; F Lean mass (%) liraglutide; G Lean mass (%) MAR709; H Lean mass (%) vehicle; I Lean mass (%) SOPF mice; J Lean mass (%) colonized mice; n = 8–9, p < 0.05∗, p < 0.01∗∗, and p < 0.001∗∗∗, n.s. = not significant.

Treatment with diabetes medication had no effect on bacterial colonization (Table 1). Body weight decreased significantly in the drug-treated groups compared to vehicle, with no difference based on the infection status. Compared to baseline, the SOPF liraglutide group lost 10.7% body weight by the end of the treatment, the colonized liraglutide group lost 12.0%, the SOPF MAR709 group lost 15.5% and the colonized MAR709 group lost 13.5%, while the SOPF vehicle group lost 1.2% and the colonized vehicle group lost 1.3% body weight. For each treatment, no differences were observed between the two hygiene statuses (Figure 2A–C). Given these results, the comparison between the different treatment groups was done combining colonized and clean mice. Liraglutide and MAR709 showed significant differences (p < 0.001) in body weight loss compared to vehicle on each day of treatment, no significant differences were seen between the drugs (Figure 2D).The comparison of the hygiene statuses within each treatment for food intake showed no statistically significant differences (Figure 2E–G). The drug-treated groups, aggregating the two infection statuses, showed reduced food intake relative to vehicle treated controls. However, no statistically significant differences were observed between the drugs (Figure 2H). Changes in body composition after treatment were greatest in MAR709, particularly in fat mass. The SOPF liraglutide group lost 19.73% fat mass, the colonized liraglutide group lost 20.91%, the SOPF MAR709 group lost 22.94% and the colonized MAR709 group lost 24.46%, while the SOPF vehicle group lost 2.51% and the colonized vehicle group gained 0.33% fat mass. Considering each treatment group separately, no differences were observed between colonized and clean mice (Figure 3A–C, 3F-H). The treated groups lost significantly more fat and lean mass compared to the vehicle (Figure 3D,E,I,J). There were no statistically significant differences between liraglutide and MAR709.

Glucose tolerance was assessed in all groups with an ipGTT. Both liraglutide and MAR709 showed significantly lower glucose levels compared to vehicle controls. Significant differences between liraglutide and MAR709 were observed at time point 0 (comparison SOPF groups), 15 min after injection (comparison SOPF groups) and 30 min after injection (comparison colonized groups). Mice in the MAR709 groups had the lowest basal glucose levels, approaching basal levels 120 min after glucose administration. Vehicle groups showed the highest glucose excursions. The two hygiene statuses had similar glucose levels, and that for each treatment separately (Figure 4A–C). Statistically significant differences were observed only for the basal blood glucose level for MAR709 (p = 0.047) and vehicle (p = 0.010). No significant differences were observed for liraglutide. Calculation of the area under the curve (AUC) showed significant differences between the drugs and vehicle, but also between liraglutide and MAR709 for both hygiene statuses (Figure 4D–H).

### 3.3 Macro- and microscopic changes resulting from exposure to HFD or bacterial colonization

Greasy fur was seen in two cages where mice had dragged the high-fat diet into the cage. One mouse had two small abscesses on its abdomen. These were thought to be due to bite wounds between males. None of the mice showed dermatitis due to the high-fat diet or clinical signs of a bacterial infection. No other abnormalities where seen in fur or skin.

**Figure 5:**
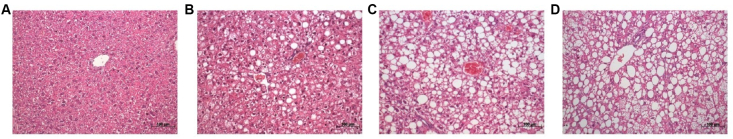
**Steatosis scores according to Brunt et al. and Kleiner et al.** [44,45]. A Score 0 normal; B Score 1 mild; C Score 2 moderate; D Score 3 severe.

Histology revealed hepatic steatosis in half of the animals in both hygiene groups (24 out of 52 mice; Figure 5 and Table 3). A chi-square test was statistically significant with a p-value < 0.05. This indicates an influence of the bacterial status on the development of steatosis. In addition, 39 mice showed periportal interstitial lymphocytic infiltrates or focal mixed cell infiltrates of lymphocytes and neutrophils. Most of the foci measured less than 100 μm in size, three mice showed larger inflammatory foci (two SOPF and one colonized). One mouse of each hygiene group also showed multifocal cell group necrosis. Mild exocrine pancreatitis with foci of lymphocytic infiltration was observed in two mice in the SOPF group. Autophagic vacuoles were found in the acinar cells of four mice in the colonized group. No other pancreatic abnormalities were observed. Renal specimens from 41 mice showed suburothelial lymphocytic infiltration of the renal pelvis (22 SOPF, 19 colonized). The duodenum samples of half of the animals in both hygiene groups showed villous fusion, focal to multifocal apical villous necrosis, follicular formation, villous apical vacuolization, excessive lymphoplasmacytic infiltration of the lamina propria and dilatation of the central villous canals. Giemsa staining revealed the presence of *H. hepaticus* in all parts of the intestine of colonized mice. No bacteria were detected in the liver. There were no abnormalities in the lungs, spleen, stomach, cecum, or colon.

**Table 3:** Steatosis liver (macrovesicular).

	SOPF	Colonized	Total
Score 0	8	20	28
Score 1	4	1	5
Score 2	8	2	10
Score 3	6	3	9
Total	26	26	52

## 4 Discussion

Microorganisms play an important role in health, especially the gut microbiome is becoming an important area of study. Changes in the composition can exacerbate, trigger and cure diseases [66]. The development of obesity and type 2 diabetes can also be exacerbated by altered intestinal flora [[38], [39], [40], [41]]. Not only in humans, but also in mice the microbiome should be considered in disease development [67]. All mice of this study originated from one breeder of the same barrier and with the same hygiene status. The microbiome should thus be very similar, as the starting situation was identical for all mice. Differences due to bacteria are attributable to the experimental infection. Stool samples demonstrated that the infected mice remained colonized with *H. hepaticus, R. pneumotropicus*, and *S. aureus* until the end of the experiment whereas the SOPF mice tested negative at all times in real-time PCR. A limitation of the study is the clearly lower copy numbers in the fecal PCR for *S. aureus* and *R. pneumotropicus* compared to those of *H. hepaticus*. This raises the question of the degree of colonization for these two bacteria and whether clinically relevant infections were achieved via oral gavage which could possibly influence the metabolic response to high-fat diet or incretin-analogues in mice to the same extent as the infection with *H. hepaticus*. One possible explanation for the large difference in copy numbers could be the preferred colonization site of the different pathogens. *H. hepaticus* colonizes exclusively the intestine, especially the cecum and colon. *R. pneumotropicus* and *S. aureus* also colonize other mucous membranes, such as the nasopharynx or genital tract [14,27,28,30,59]. Nevertheless, similar copy numbers of stool samples can be found in the literature for *R. pneumotropicus* [68]. Furthermore, it was demonstrated that mice colonized by *R. pneumotropicus* were tested positive by PCR using fecal pellets over a period of one year [69]. To ensure that the infection route via oral gavage leads to a stable colonization with all three bacterial species used and that fecal pellets are suitable for confirming the infection, a preliminary test was performed on six mice in advance. In addition to the analysis of fecal samples with PCR, swap samples from different body regions showed bacterial growth on Columbia blood agar plates four weeks after experimental infection. *R. pneumotropicus* was present in all mouth-and-throat swabs as well as nose swabs and in some smears of the preputium and the eyes. All mice showed bacterial growth for *S. aureus* in mouth-and-throat and preputial swabs and occasionally in swabs from the nose. Therefore, samples from other parts of the body than feces could have achieved higher copy numbers in real-time PCR. Based on the results of the preliminary test and positive PCR results over the entire testing period, we assume successful colonization with all three bacteria in all experimentally infected mice. At the same time, we cannot exclude the fact that other routes of infection would have been more suitable for *R. pneumotropicus* and *S. aureus* to achieve a higher degree of colonization and thus a possibly greater potential impact throughout the experiment.

As body weight gain and food intake were similar in both hygiene groups, the model can be used either with or without the presence of *H. hepaticus, R. pneumotropicus* and *S. aureus*. Changes in blood values due to the development of pre-diabetes were seen in increased insulin levels in both hygiene groups. In colonized mice, insulin levels were lower compared to SOPF. Cells were possibly less insulin resistant in colonized mice. Contrary results can be found in the literature for *S. aureus*, where colonization causes the production of an insulin-binding protein that leads to increased insulin resistance [70]**.** In the ipGTT, no differences in glucose tolerance were observed between the colonized mice and the SOPF mice for each treatment. The drug efficacy test was not adversely affected by colonization. Glucose levels remained constant throughout the experiment, presumably the increased insulin levels compensated for the insulin resistance and increasing blood glucose. This was also observed in the data of Schile in DIO mice [71]. Total cholesterol levels were fluctuating upwards and downwards in both groups during the feeding phase. At the last measurement for cholesterol, SOPF mice had slightly higher levels compared to the first measurement at 4 weeks, while colonized mice had slightly lower levels, with a statistically significant difference between the two groups at this time point. Like cholesterol, TAG levels were oscillating between the first and last measurements. The colonized group showed slightly higher levels after 24 weeks of high-fat diet, two time points showed significant differences with higher levels for SOPF (week 12 and 20). We assume that these differences are natural fluctuations, as they were only seen for single time points [72]. To summarize the metabolic changes during the development of pre-diabetes, no significant differences were seen between the two hygiene groups at the last measurement before treatment, except for cholesterol.

Drug efficacy was not affected by bacterial colonization. For liraglutide, MAR709 and vehicle, no differences were observed between the hygiene groups regarding body weight loss, food intake and changes in body composition during drug administration. Food intake and body weight loss were significantly lower in the treated groups compared to vehicle. Body composition also showed a significant loss of fat mass with both treatments compared to vehicle. MAR709 induced the greatest loss of fat mass and body weight, as seen in the studies by Finan et al. and Zhang et al. [52,53]. Impaired glucose tolerance is a hallmark of pre-diabetes. Therefore, an ipGTT was performed after the treatment week. There were significant differences for the basal glucose levels of MAR709 and vehicle comparing SOPF against colonized groups. During the ipGTT, no significant differences in hygiene status were found within the drug and vehicle groups. Therefore, the baselines differences can be neglected. MAR709 treated groups achieved the lowest basal glucose levels and lowest glucose excursions. The AUC showed significant differences in glucose tolerance between drugs and vehicle and also between MAR709 and liraglutide. This confirms the superiority of the co-agonist over the mono-agonist in the treatment of diabetes.

Since mice of both hygiene groups were affected by liver steatosis, it is likely that the observed changes are diet related. Fatty liver changes are common in overweight patients [73]. Steatosis to steatohepatitis are known effects of an unhealthy diet in mice [[74], [75], [76], [77]]. Liver inflammation and steatosis may also be caused by colonization with *H. hepaticus.* The used mouse strain C57BL/6J is immunocompetent and is regarded as resistant to clinical symptoms under *H. hepaticus* colonization [14,24,25]. The main colonization site is the cecum, which was confirmed in a pilot study [14,59,78]. No bacteria were observed in the Giemsa stain of the liver. We assume that the cause of steatosis to steatohepatitis is HFD feeding. The significant chi-square test indicates a positive influence of the bacterial status on the development of steatosis. Many studies have investigated the influence of the microbiome on the development of non-alcoholic fatty liver disease [[79], [80], [81], [82], [83], [84]]. In contrast to our observations, where colonized mice showed a lower incidence, in literature an increased steatosis under bacterial colonization with *S. aureus* is reported. Bacterial overgrowth of *Staphylococci* was found in the small intestine in patients with non-alcoholic fatty liver steatosis [85]. Experimental infection with *H. hepaticus* in susceptible BALB/c mice caused hepatitis and can intensify steatosis under HFD feeding [86,87]. In wildtype C57BL/6 mice, it has been shown that *Helicobacter* colonization can have a positive influence on inflammatory processes. After experimental infection, anti-inflammatory IL-10 production was increased as well as transcription factors to downregulate T-cell activation. In addition, genes for cell repair were activated [88]. These positive effects of inflammation downregulation could be the reason for lower steatosis levels in colonized mice in our study. Changes in intestinal villi length and lesions are also effects of HFD feeding [89,90]. Fat vacuolization and inflammation have been described in the literature [91,92]. The abnormalities of the pancreas can be neglected because they occurred only in the exocrine part. Mild inflammation in the area of the renal pelvis was seen in both hygiene groups. This is a common secondary finding in male mice housed together.

The colonization with the three bacteria did not impact the development of pre-diabetes and treatment in the DIO model in immunocompetent C57BL/6 mice. Nevertheless, effects of bacterial colonization with these agents may occur in immunosuppressed organisms. *H. hepaticus* causes inflammatory bowel disease in IL-10-deficient mice [93,94]. This could have major implications for the model as colitis increases under HFD [[95], [96], [97]]. An interleukin-10 knockout could also influence staphylococcal infection. *S. aureus* manipulates the immune system by enabling colonization through the stimulation of anti-inflammatory IL-10 production [98]. Higher IL-10 levels were also found in HFD fed mice compared to lean mice in a sepsis model. HFD adversely affects the immune system and lead to higher mortality in this study [99]. *S. aureus* infection is also proinflammatory in adipose tissue [100]. The defense mechanisms against the bacteria depend on the innate immune system [22]. Additional immunosuppression could increase clinical infection caused by *S. aureus* and may influence the DIO model. The same applies to *R. pneumotropicus* that shows no clinical infection in immunocompetent but in immunodeficient mice [20,21].

## 5 Conclusions

Our results show that the DIO model under bacterial colonization with *H. hepaticus, R. pneumotropicus*, and *S. aureus* can be used for pre-diabetes studies and drug evaluation. In conclusion, no major differences were observed between colonized mice and SOPF mice. Treatment with incretin-analogues showed similar results and superior performance in terms of glucose tolerance for the new compound MAR709 compared to liraglutide. It should be noted that these results were obtained in the DIO model using male C57BL/6J mice. They may not be equally transferred to all diabetes models, to conventionally housed mice, to immunodeficient strains or colonization with other bacterial pathogens, but the results provide an indication that experiments do not have to be performed exclusively in SOPF animals. The potential effect of bacteria and altered microbiome on study results should always be considered in the experimental design.

## CRediT authorship contribution statement

**Margit Wunderlich:** Writing – original draft, Visualization, Investigation. **Manuel Miller:** Writing – review & editing, Supervision, Investigation, Conceptualization. **Bärbel Ritter:** Investigation. **Ronan Le Gleut:** Writing – review & editing, Formal analysis. **Hannah Marchi:** Writing – review & editing, Formal analysis. **Monir Majzoub-Altweck:** Writing – review & editing, Investigation. **Patrick J. Knerr:** Resources, Methodology. **Jonathan D. Douros:** Resources, Methodology. **Timo D. Müller:** Writing – review & editing, Resources, Formal analysis, Conceptualization. **Markus Brielmeier:** Writing – review & editing, Supervision, Project administration, Conceptualization.

## Declaration of competing interest

The authors declare that they have no known competing financial interests or personal relationships that could have appeared to influence the work reported in this paper.

## Data Availability

Data will be made available on request.
